# Implementation of a Numerically Stable Algorithm for Capillary Hysteresis in Gas Hydrate Deposits

**DOI:** 10.1021/acs.energyfuels.4c01516

**Published:** 2024-07-10

**Authors:** Jihoon Kim, Hyun Chul Yoon

**Affiliations:** † Harold Vance Department of Petroleum Engineering, Texas A&M University, 3116 TAMU Richardson Building, College Station, Texas 77843, United States; ‡ Marine Geology & Energy Division, Korea Institute of Geoscience and Mineral Resources (KIGAM), 124 Gwahak-ro, Daejeon 34132, Republic of Korea

## Abstract

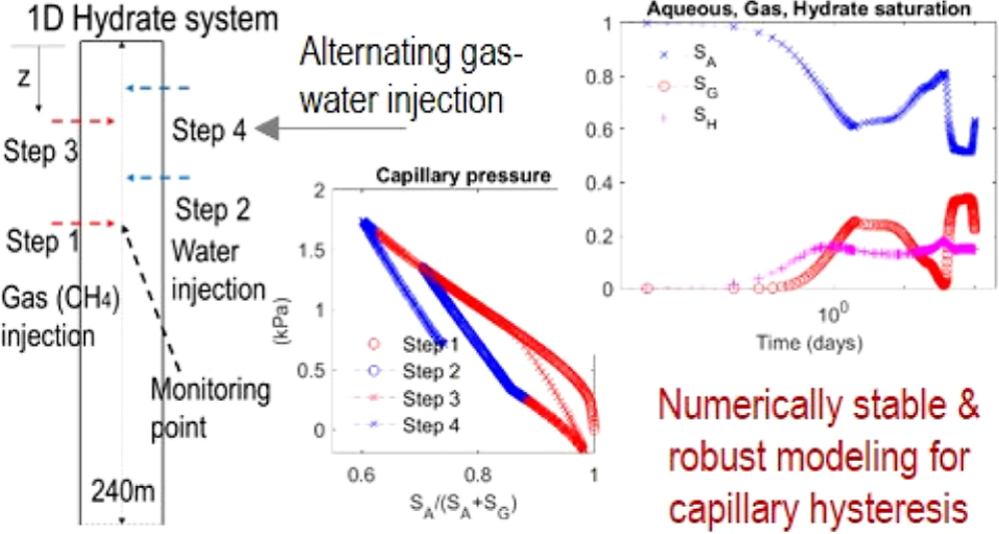

We develop a numerically stable algorithm of intrinsic capillary hysteresis for numerical simulation of gas hydrate deposits where cyclic drainage and imbibition processes occur. The algorithm is motivated by the elastoplastic return mapping, and it is an extension of the recently developed algorithm of two-phase immiscible flow, which provides numerical stability with the fully implicit method. We consider the effective gas and aqueous saturations normalized by total fluid phase saturation implicitly affected by the dynamic formation and dissociation of hydrates. Specifically, gas saturation is additively decomposed into the reversible and irreversible parts, and the algorithm computes the reversible and irreversible parts dynamically during the evolution of gas saturation. We perform numerical tests, including a field-scale case, by implementing the code of the capillary hysteresis in a gas hydrate flow simulator. We find that the developed algorithm is stable and robust for repeated drainage and imbibition processes in gas hydrate systems. Since cyclic depressurization is one of the promising production scenarios for gas production from marine gas hydrate deposits, the developed algorithm and code will provide robust and high-fidelity simulation in the forward simulation of multiphase flow.

## Introduction

Subsurface engineering problems encountered in gas hydrate deposits, CO_2_ sequestration, groundwater contamination, and oil/gas reservoir development, invariably entail multiphase flow phenomena in porous media, where capillarity emerges as a fundamental determinant.^1−5^ In partially saturated porous media, matrix suction exerts a significant influence on soil mechanics, subsurface deformation, effective stress regimes, and multiphase fluid dynamics.^6^ Furthermore, fracture capillary pressure mechanisms play a pivotal role in the behavior of multiphase flow in fractured rock formations, necessitating comprehensive efforts in physical interpretation, mathematical formulation, and computational simulation to elucidate the underlying processes.^5^ A multitude of investigations have been conducted to discern the intricate interplay between capillary pressure dynamics and shifts in the hydrate phase in gas hydrate deposits.^7−11^ Notably, empirical findings by Yan et al.^12^ have underscored the propensity for heightened hydrate saturation to elevate the capillary pressure curve.

Furthermore, cyclic imbibition and drainage processes often occur in multiphase flow in porous media due to various injection–production scenarios or gravity effects.^13−17^ Also, cyclic depressurization at UBGH2–6 in the Ulleung Basin is one of the promising production scenarios, which can maximize gas production but minimize subsidence.^18^ When a cyclic bottom hole pressure condition is applied to the reservoir, wetting and non-wetting phase saturations change periodically and non-monotonically. As a result, cyclic imbibition and drainage processes in the presence of multiple fluid phases can cause irreversible flow, which yields history-dependent capillary pressure and relative permeability.^19,20^ Capillary hysteresis exists in multiphase flow in porous media, affecting the residual saturation of gas and relative permeability. When the capillary hysteresis cannot be modeled, the amount of trapped gas and the flow capacity cannot be estimated reliably, and thus, we would not predict productivity of gas appropriately. Even though the hysteresis of the capillary pressure and relative permeability can play an important role in the cyclic changes in the saturation, the hysteresis modeling associated with gas hydrate deposits has not sufficiently been investigated.

Several studies show the importance of the numerical modeling for hysteretic behavior in multiphase flow,^4,21−24^ but it is challenging to keep numerical stability during several cyclic imbibition and drainage processes. Capillary hysteresis modeling fails frequently for cyclic drainage–imbibition processes when the numerical method is simply based on flags that indicate drainage and imbibition, although it is intuitive and simple but ad hoc. To fix the numerical instability, a more robust formulation and numerical method is required to be consistent with the thermodynamics laws, particularly the second law of thermodynamics, which can yield well-posed mathematical problems.

In particular, the hysteresis modeling for the gas hydrate deposits becomes much more difficult because the changes of the solid phase need to be taken into account for capillarity simultaneously. Two apparent hysteretic behaviors can be observed in gas hydrate deposits. One is capillary hysteresis induced by changes in the pore structure/volume as well as formation or dissociation of gas hydrates. The other is intrinsic capillary hysteresis typically shown in multiphase flow in porous media. Both of them are related to solid phase structures. Yoon and Kim^11^ studied the impact of pore volume changes and scaling effects induced by hydrate dissociation/formation as well as geomechanics on capillary hysteresis, while the intrinsic capillary hysteresis was not considered.

In this study, we emphasize numerically stable simulation of the intrinsic capillary hysteresis, which yields a reasonable solution without numerically severe spurious oscillation in time. Specifically, we employ a recently developed algorithm based on elastoplastic return mapping.^25^ Just as the total strain is decomposed into elastic and plastic strains, the fluid content (saturation) can similarly be decomposed into reversible and irreversible parts. Note that the constitutive relations of hysteresis in this study generate well-posed mathematical problems, being thermodynamically consistent.^25^ Also, we introduce an effective fluid saturation, normalized by the total fluid phase saturation because the hydrate saturation changes due to dissociation and formation repeatedly. We implement the numerical code of capillary hysteresis in the TOUGH + HYDRATE simulator.^26^

From numerical simulation, we identify numerical stability during repeated imbibition and drainage processes, where the capillary pressure curves are hysteretic, exhibiting irreversibility. During the evolution of capillary pressure, we will find some combined effects from hydrate formation and dissociation of gas hydrates due to the thermodynamic behavior. We show two numerical examples of capillary hysteresis in gas hydrate deposits. We first take a 1D test case to test the numerical behavior of the algorithm and implemented code, where two capillary pressure models are considered: the semilog model and the van Genuchten model. Then, we apply it in a field-scale case, a site of UBGH2-6 in Ulleung Basin, South Korea,^18^ where the van Genuchten model is used. Because this algorithm is robust and similar to elastoplastic geomechanics, it can be straightforwardly extended to coupled flow and geomechanics of gas hydrate deposits.

## Mathematical Formulation

We restate the governing equation of non-isothermal multiphase flow related to methane hydrate deposits.^26^ The equation for fluid flow is based on the mass balance
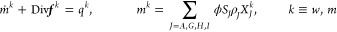
1where superscript *k* indicates the fluid component. *m*^*k*^, ***f***^*k*^, and *q*^*k*^ are its mass, flux, and source terms, respectively, and subscript *J* indicates the fluid phase. Div(·) is the divergence operator, and  denotes the time derivative of a physical quantity of (·). ϕ, *S*_*J*_, ρ_*J*_, and *X*_*J*_^*k*^ are the porosity, saturation, density of phase *J*, and mass fraction of component *k* in phase *J*, respectively. There are four possible phases *J*: aqueous (*J* = *A*), gaseous (*J* = *G*), hydrate (*J* = *H*), and ice (*J* = *I*). Only two components {i.e., water [H_2_O (*k* = *w*)] and methane [CH_4_ (*k* = *m*)]} are considered, and the hydrate phase is considered as one possible phase of the CH_4_·H_2_O system. ***f***^*k*^ is stated as
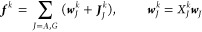
2where ***w***_*J*_^*k*^ and ***J***_J_^k^ are the convective and diffusive mass fluxes of component *k* in phase *J*, respectively. The convective flow is described by the Darcy’s law
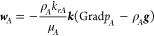
3
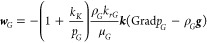
4where Grad(·) is the gradient operator, ***k*** is the absolute permeability tensor, and *k*_*K*_ is the Klinkenberg factor for gas flow. μ_*J*_, *k*_*rJ*_, and *p*_*J*_ are the viscosity, relative permeability, and pressure of phase *J*, respectively.

The diffusive flow is described by Fick’s law as
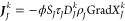
5where *D*_*J*_^*k*^ and τ_*J*_ are the hydrodynamic dispersion coefficient and tortuosity of phase *J*, respectively.

The governing equation for heat flow can be obtained from the energy balance^26^
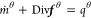
6where superscript θ indicates the heat component. *m*^θ^, ***f***^θ^, and *q*^θ^ are heat, flux, and source terms, respectively. The heat accumulation term, *m*^θ^, is expressed as
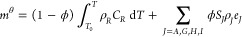
7where ρ_*R*_, *C*_*R*_, *T*, and *T*_0_ are the density and heat capacity of porous media, temperature, and reference temperature, respectively. *e*_*J*_ is the specific internal energy for phase *J*.

The heat flux is
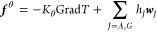
8where *K*_θ_ is the composite thermal conductivity of porous media and *h*_*J*_ is the enthalpy of phase *J*. The specific internal energy and enthalpy for phase *J* are written as
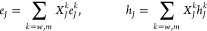
9

**Figure 1:**
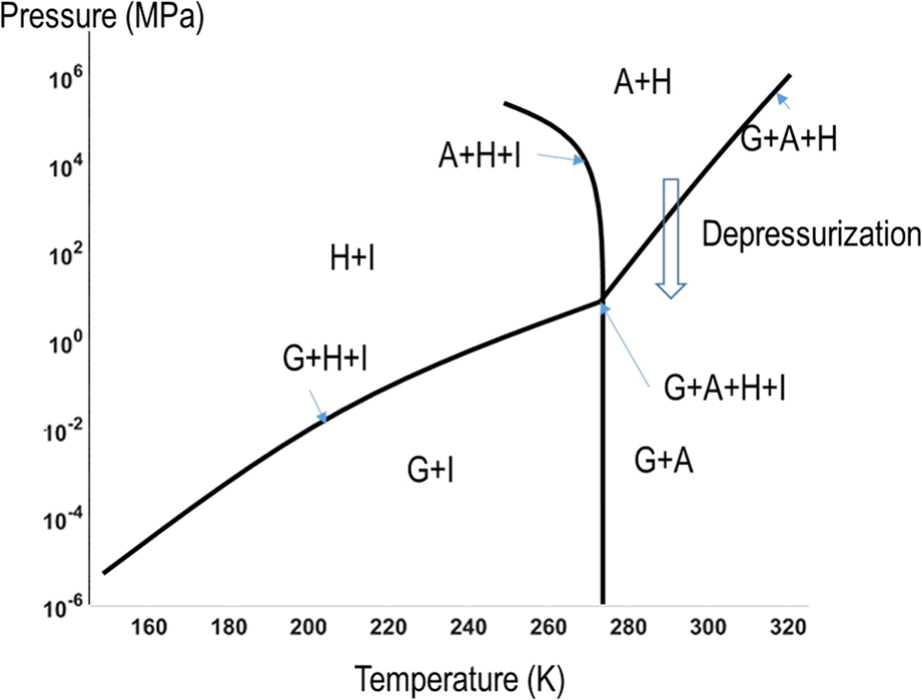
Pressure–temperature equilibrium relationship of the aqueous (A)–gas (G)–hydrate (H)–ice (I) system.

Dissociation and formation of methane hydrates are described as
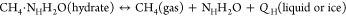
10where *N*_H_ is the specific hydration number of the methane hydrate and *Q*_H_ is the enthalpy of hydration/dissociation. Considering the equilibrium condition, the heat of the hydrate dissociation is taken into account when differentiating the hydrate mass between two points in time, described as
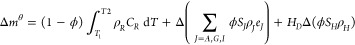
11where *T*_1_ and *T*_2_ are the temperatures at these two points in time and *H*_*D*_ is the heat of hydrate dissociation.^26^ The specific reaction of eq 10 is modeled based on the equilibrium relationship in the phase diagram shown in Figure 1. The hydrates are dissociated when the pressure decreases, which motivates a methane production method such as depressurization.

For relative permeability, we take the modified version of Stone’s relative permeability model, written as
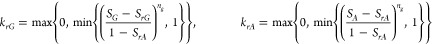
12where we take *n*_*g*_ = 4,*S_rG_* = 0, and, *S_rA_* = 0.15 unless otherwise noted.

## Capillary Hysteresis and Its Numerical Implementation

**Figure 2:**
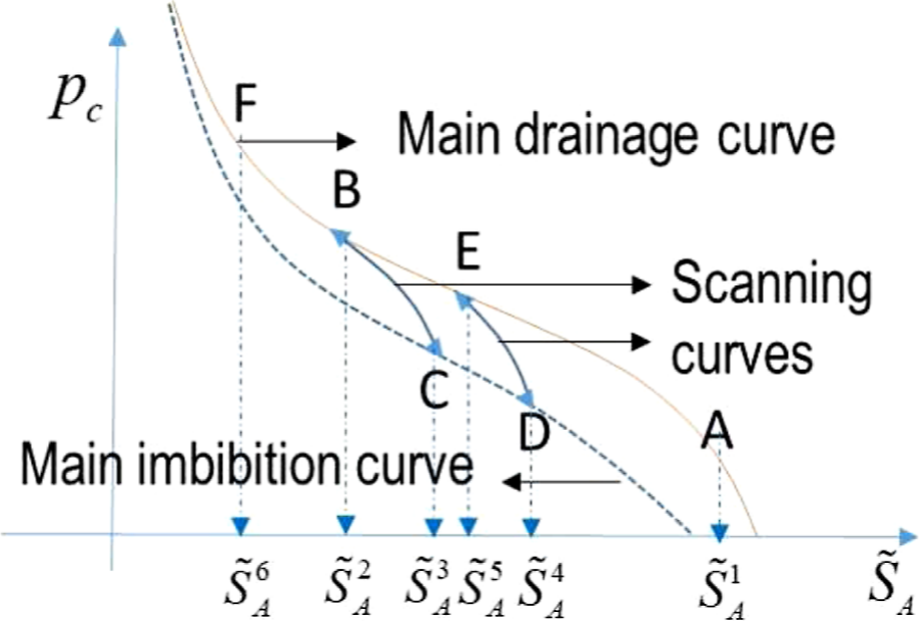
Schematic capillary pressure curve of hysteresis.

Capillary pressure between two immiscible phases such as gas and aqueous phases is defined as
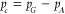
13where gas and aqueous phases are nonwetting and wetting phases, respectively. Capillary pressure is a function of saturation [i.e., *p*_*c*_(*S*_*J*_)], and it frequently shows history-dependent behavior during imbibition and drainage processes, known as capillary hysteresis. In other words, *p*_*c*_ can have different values even if the saturation is the same (Figure 2). For example, when a path of  is taken as shown in the figure, the corresponding path of *p*_*c*_ becomes *A* → *B* → *C* → *D* → *E* → *F*, where *A* → *B*, *B* → *C*, *C* → *D*, *D* → *E*, and *E* → *F* are irreversible drainage, reversible imbibition, irreversible imbibition, reversible drainage, irreversible drainage processes, respectively. Instead, when a different path of  is taken, the corresponding path of *p*_*c*_ is *A* → *B* → *C* → *B* → *F*, where *C* → *B* is reversible drainage. Thus, this capillary hysteresis is fundamentally an irreversible physical process similar to elastoplasticity.

Nuth and Laloui^23^ proposed an algorithm for hysteretic capillary pressure, motivated by the modeling of elastoplastic mechanics, and Yoon et al.^25^ further studied mathematical analyses of contractivity and algorithmic stability for two-phase flow systems followed by numerical tests. Here, we extend the original ideas to gas hydrate systems in which solid and mobile phases interact with each other. Gas and aqueous saturations are required to be normalized by mobile saturation, and they are decomposed into the reversible and irreversible parts additively, as follows.
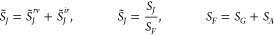
14where superscripts *rv* and *ir* indicate reversible and irreversible saturations of phase J, respectively, and *S*_*F*_ is the total fluid saturation. Here, we focus on the behavior of gas saturation, where *S*_*G*_^*ir*^ is changed dynamically, while *S*_*A*_^*ir*^ is assumed to be unchanged, being the maximum.

Since , we state the relationship between capillary pressure and reversible gas saturation, expressed in a rate form, as
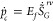
15where *E*_*f*_ is a positive capillary modulus for reversible saturation just like elastic mechanics.

For the modeling of irreversible saturation, we introduce the yield function and the relation between hardening variables, written as
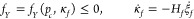
16where *f*_*Y*_ and κ_*f*_ are a yield function and a pressure-like hardening variable, respectively. *f*_*Y*_ is defined as a convex function for a well-posed problem.^27^ ξ_*f*_ and *H*_*f*_ are saturation-like hardening variables and positive hardening moduli for capillary hysteresis, respectively. To characterize the evolution of , the associated flow rule is taken as
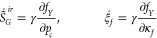
17where γ is an irreversibility multiplier. Additionally, the Kuhn–Tucker and consistency conditions are required, written as
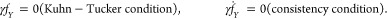
18

Referring to Yoon et al.,^25^ we estimate that the formulation for capillary hysteresis can provide well-posedness (i.e., contractivity) for gas and aqueous flow systems, provided that the governing equations of gas hydrate problems are well-posed. Thus, its numerical implementation in an existing gas hydrate simulator (e.g., TOUGH + Hydrate) yields numerical stability when the fully implicit method is used. For space discretization, we take the finite volume method with the piecewise constant interpolation of the pressure, saturation, and temperature. The Newton–Raphson method is employed to solve nonlinear problems.

Let us implement the constitutive relations of capillary hysteresis described above by taking the concept of return mapping more specifically. In order to avoid confusion of terminology and notation across the literature, it is worth noting that plastic water saturation *S*_*w*,*p*_ in the previous study^25^ corresponds to irreversible gas saturation S_*g*_^*ir*^ in this study.

We consider two types of capillary pressure curves: a semilog function with an entry pressure and the van Genuchten model, expressed as follows. The specific algorithms are described in Algorithms 1 and 2.
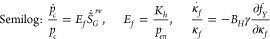
19
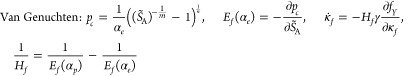
20where *p*_*en*_ is the entry capillary pressure and *K*_*h*_ is a positive modulus for the reversible process. *B*_*H*_ is a positive modulus that characterizes the evolution of hardening parameters. α_*e*_ and α_*p*_ are positive moduli for the reversible and irreversible processes, respectively. . For the yield functions, we take the forms as
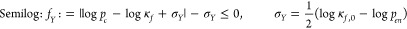
21
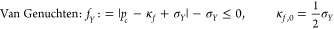
22where σ_*Y*_ is a constant that limits the reversible domain of capillary pressure. κ_*f*,0_ is an initial value of κ_*f*_.

**Figure 3:**
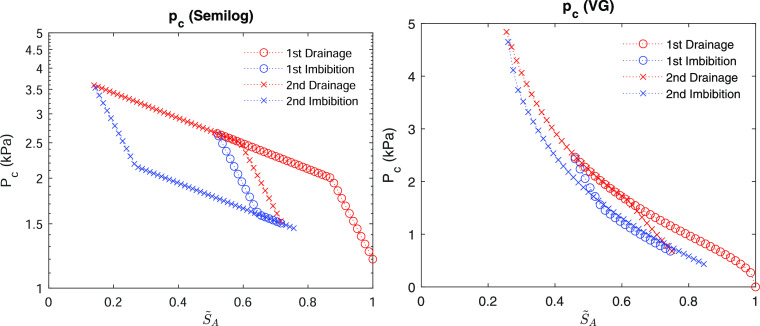
Left: the semilog model. Right: the van Genuchten model.

Figure 3 shows the evolution of capillary pressure during repeated drainage and imbibition processes from eqs 19 and 20 and Algorithms 1 and 2, where two fluid phases exist. The semilog model takes *p*_*en*_ = 1.2 kPa, *K*_*h*_ = 4.8 kPa, *B*_*H*_ = 1.0, and κ_f,0_ = 2.0 kPa. The van Genuchten model takes α_*e*_ = 10^–4^ Pa^–1^, α_*p*_ = 2 × 10^–4^ Pa^–1^, *n* = 2.0, and σ_*Y*_ = 0.5 kPa. We identify from the figure that the numerical algorithms can simulate cyclic drainage and imbibition behavior well and stably. We then implement these algorithms in a gas hydrate flow simulator, specifically TOUGH + Hydrate, to model capillary hysteresis.^26^ Note that one may select another gas hydrate simulator such as STOMP.^28^ Here, the hardening modulus for the semilog model is constant in order to be consistent with Nuth and Laloui,^23^ where it is based on a laboratory experiment. On the other hand, the hardening modulus for the van Genuchten model is not constant but dynamic during simulation in order to follow the main drainage and imbibition curves. Hence, one can opt for either a constant or dynamic hardening modulus depending on the specific capillary pressure model employed.
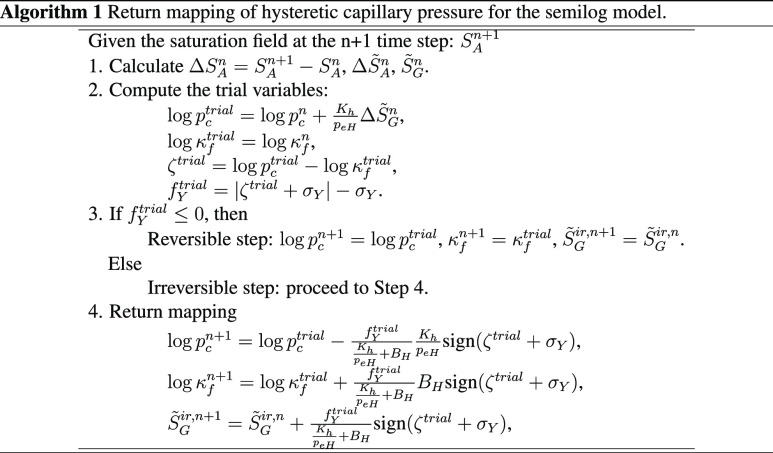

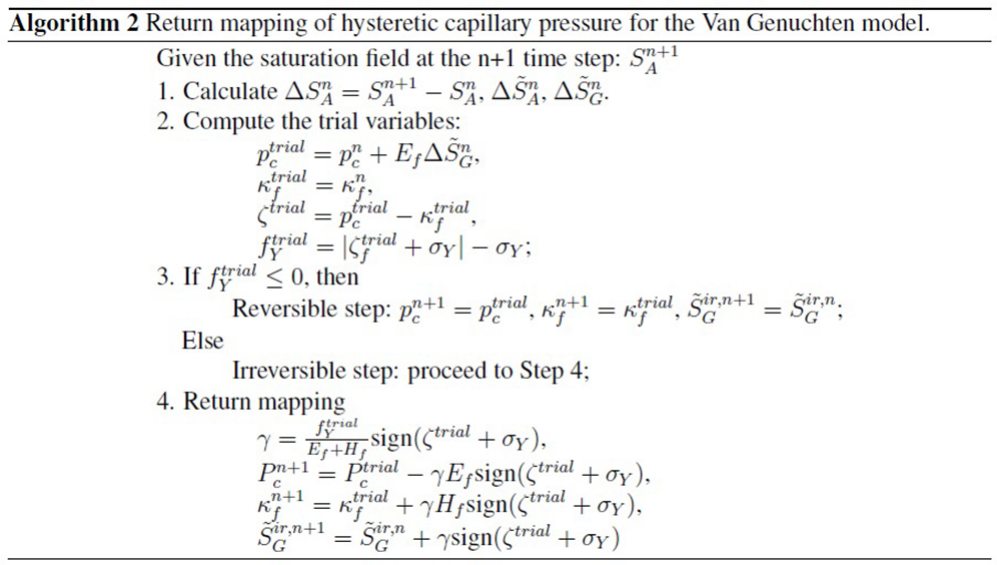


## Numerical Experiments

**Figure 4:**
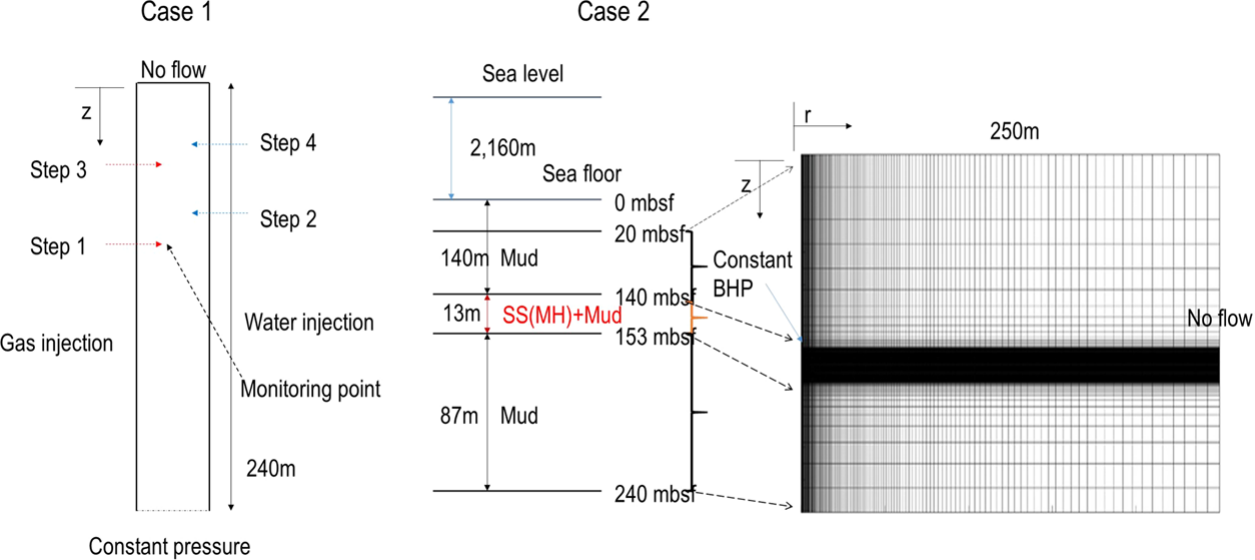
Schematic representation of Cases 1 and 2.

We consider two test cases: a 1D synthetic problem to test the numerical behavior of the implemented code and a field-scale problem to test the applicability of the implemented code in marine gas hydrate deposits. The domains of numerical simulation are shown in Figure 4. Case 1 is similar to the test problem in Yoon et al.^25^ except for the boundary condition and injection scenario. Case 2 is a field-scale problem of a gas hydrate deposit at UBGH2-6 located in South Korea.

### Case 1: Repeated Drainage and Imbibition in 1D

The domain of *L*_*z*_ = 240*m* is discretized with a uniform grid block (Δ*x* = 10*m*, Δ*y* = 10*m*, Δ*z* = 4*m*) based on the Cartesian coordinate system. We take a no-flow boundary condition at the top but a constant pressure boundary condition at the bottom with no gravity. The permeability and porosity are 1.084 × 10^–15^ m^2^ (1.1 mD) and 0.422, respectively. The bulk density is ρ_*b*_ = 2650 m^3^/kg. The initial condition is *p_A_* = 3.65 MPa, *S*_*G*_ = 0.0, *S*_*A*_ = 1.0, and *T* = 2.22 °C. Heat conductivity is 1.45 W/m/°C and rock specific heat capacity is 800 J/kg/°C. Table 1 shows injection scenarios to simulate repeated drainage and imbibition to test the two capillary pressure models. For the van Genuchten model, we additionally inject heat with a rate of 10 kW/s along with fluid injection.

**Table 1:** Injection Scenarios for Drainage and Imbibition^a^

steps	time (days)		rate (kg/s)		location (m)	fluid
	semilog	VG	semilog	VG		
1	0–2	0–2	0.2	0.1	–58	gas
2	2–10	2–20	0.1	0.2	–46	water
3	10–100	20–50	0.15	0.2	–34	gas
4	100–150	50–100	0.05	0.2	–22	water

a “Semilog” and “VG” denote the semilog model and the van Genuchten model, respectively.

**Figure 5:**
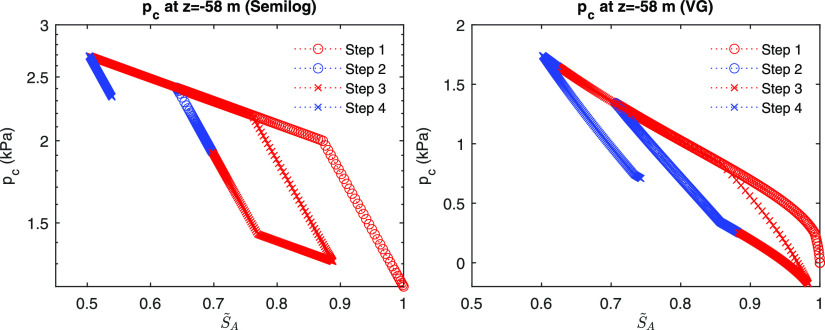
Evolution of *p*_*c*_ at the monitoring point (*z* = −58 m). Left: the semilog model. Right: the van Genuchten model.

Figure 5 shows the evolution of capillary pressure during the four steps, where drainage and imbibition occur repeatedly. For the case of the semilog model, the first drainage occurs due to gas injection at Step 1. At Step 2, we observe the first imbibition process induced by water injection. At Step 3, the imbibition occurs at the monitoring point in the early time, while *S*_*G*_ decreases because water influx is still dominant. Then, at a later time, the second drainage occurs since gas influx becomes larger than water influx after the breakthrough of gas influx. At Step 4, the second imbibition results from water injection. We observe the same behavior for the van Genuchten model. Note that the numerical results are stable despite nonmonotonic evolution of saturation, simulating capillary hysteresis well.

**Figure 6:**
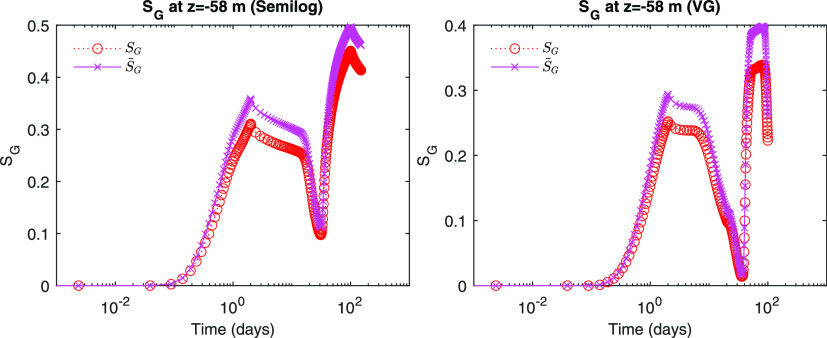
Evolution of gas saturation.

**Figure 7:**
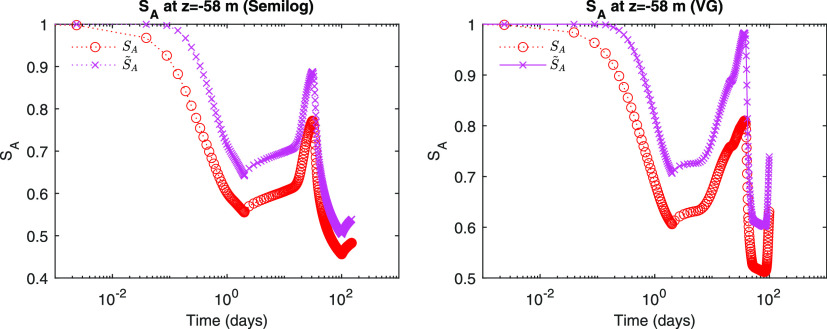
Evolution of aqueous saturation.

**Figure 8:**
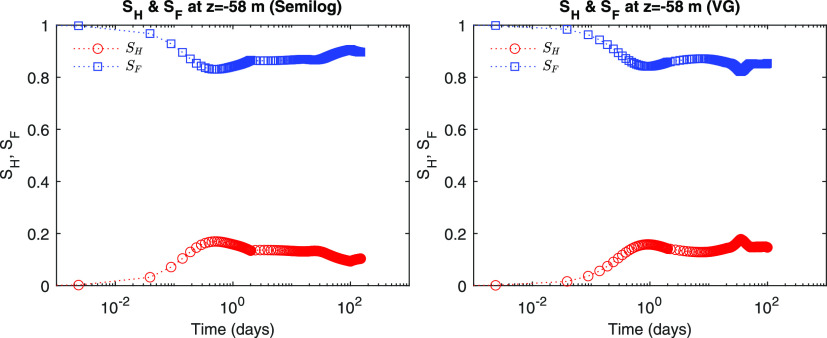
Evolution of hydrate saturation.

We identify from Figures 6 and 7 that both *S*_*A*_ and *S*_*G*_ repeatedly increase and decrease due to alternating injections of gas and water. Note that gas injection induces hydrate formation, shown in Figure 8, from which *S*_*F*_ decreases and thus  and  become higher than *S*_*G*_ and *S*_*A*_. This implies that hydrate saturation indirectly affects the intrinsic capillary hysteresis.

**Figure 9:**
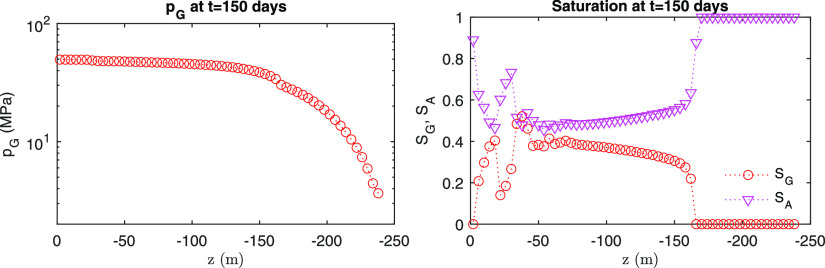
Distribution of pressure and saturation from the semilog model.

**Figure 10:**
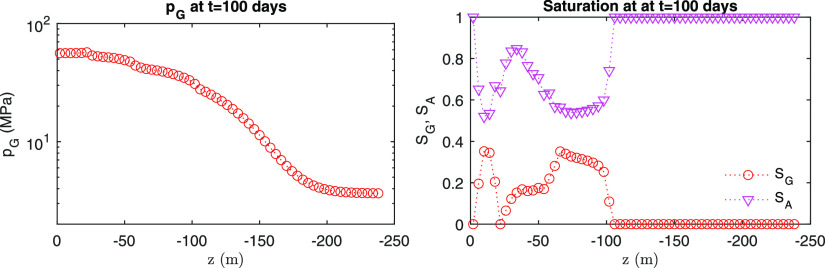
Distribution of pressure and saturation from the van Genuchten model.

From Figures 9 and 10, we also identify numerical stability at the end of the simulation for the fields of pressure and saturation. Due to continuous injection, pressure is distributed monotonically for both capillary models. Some fluctuations of saturation near the injection points are found, being based on the physics from the alternating gas and water injection during simulation. From here on, this VG model (i.e., α_*e*_ = 10^–4^ Pa^–1^, α_*p*_ = 2 × 10^–4^ Pa^–1^, *n* = 2.0, and σ_*Y*_ = 0.5 kPa) is set as the reference case unless otherwise stated for the following comparison study. The reference case only considers hysteresis in capillary pressure, but not in relative permeability.

**Figure 11:**
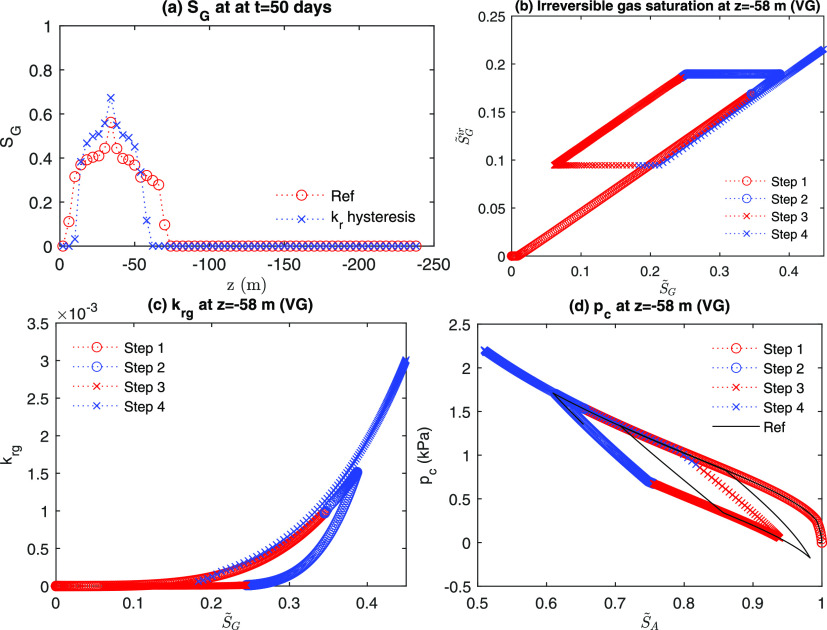
Hysteresis in gas relative permeability: (a) distribution of gas saturation at 50 days (after Step 3) and evolutions of (b) irreversible gas saturation, (c) relative permeability, and (d) capillary pressure. “Ref” denotes the reference case.

The irreversible saturation of the gas phase can be considered as residual gas saturation (i.e., *S*_*rG*_ = S_*G*_^*ir*^). Then, the relative gas permeability also becomes hysteretic and dynamic, as discussed in Yoon et al.^25^ From eq 12, the relative gas permeability decreases as the *S*_*rG*_ increases. Since S_*A*_^*ir*^ is assumed to be constant, *S*_*rA*_ is also constant. Figure 11a shows distributions of gas saturation after the second gas injection (Step 3). Gas saturation propagates more slowly in the case of the hysteretic gas relative permeability than that without the hysteretic permeability, where the initial *S*_*rG*_ is almost zero. In Figure 11b, we identify that S_*G*_^*ir*^ increases to a greater extent than the initial value when *S*_*G*_ increases during Step 1. At Step 2, even though *S*_*G*_ decreases, S_*G*_^*ir*^ does not decrease until the capillary pressure enters the main imbibition curve. As a result, this behavior affects the evolution of gas relative permeability, shown in Figure 11c, followed by the overall flow capacity of gas and aqueous phases. In turn, this physical process causes different evolutions of capillary pressure with and without hysteretic relative permeability (Figure 11d). Since we focus on numerical implementation of capillary hysteresis in gas hydrate problems, we do not perform further in-depth investigation on hysteretic relative permeability in this study, although it is one of the critical topics in reservoir engineering, which will be studied in the future.

**Figure 12:**
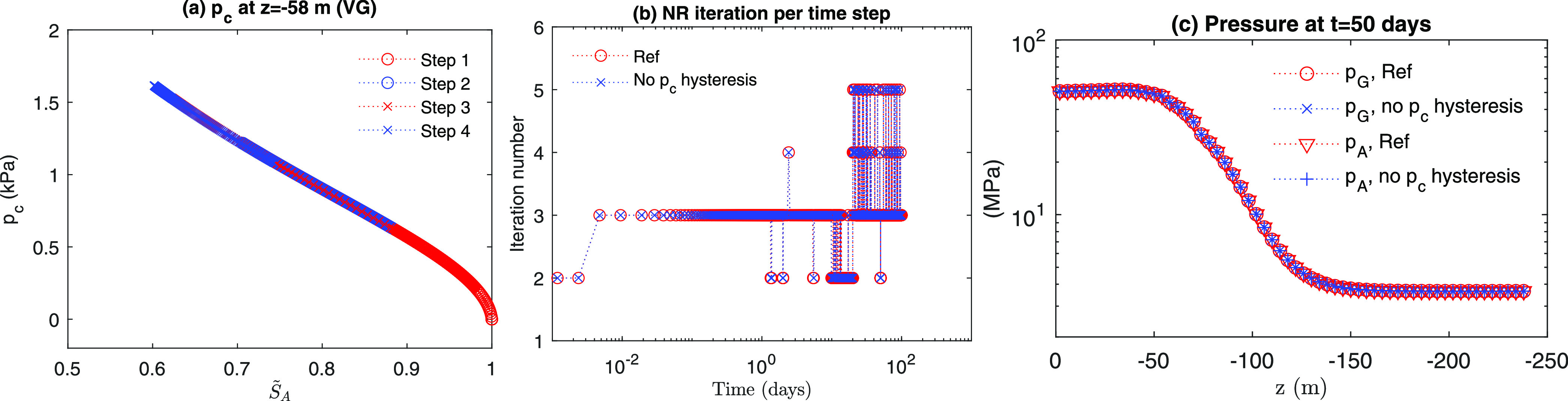
Evolutions of (a) capillary pressure and (b) number of NR iteration when α_*e*_ = 2.0 × 10^–4^ Pa^–1^ and σ_*Y*_ = 10 MPa. (c) Pressure distribution after Step 3.

**Figure 13:**
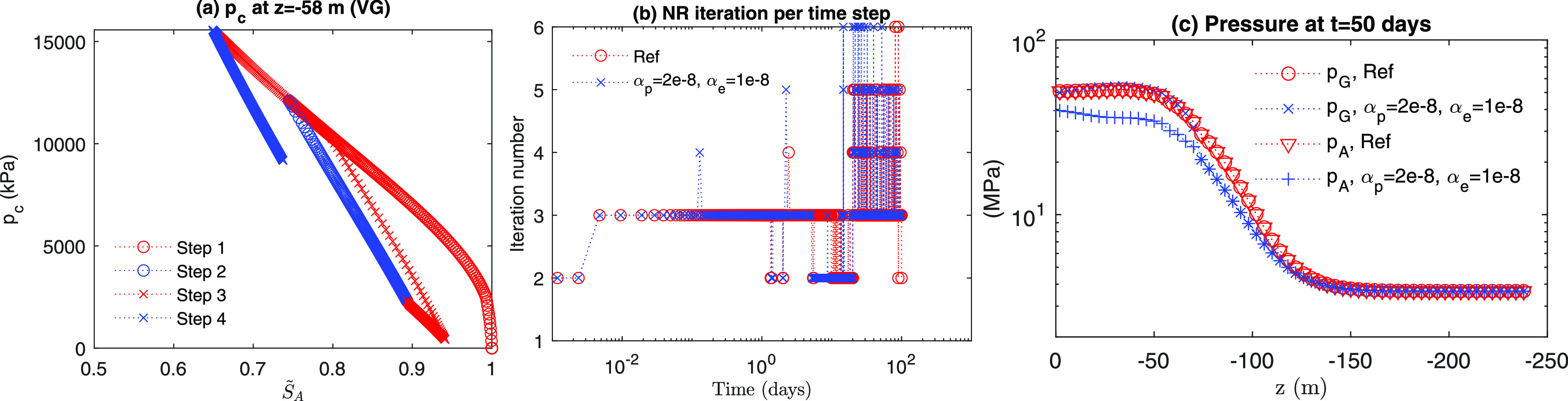
Evolutions of (a) capillary pressure and (b) number of NR iteration when α_*p*_ = 2.0 × 10^–8^ Pa^–1^, α_*e*_ = 1.0 × 10^–8^ Pa^–1^, and σ_*Y*_ = 5.0 MPa. (c) Pressure distribution after Step 3.

We investigate computational loads with and without capillary hysteresis. For no capillary hysteresis, we take α_*e*_ = 2.0 × 10^–4^ Pa^–1^ and σ_*Y*_ = 10 MPa to ensure reversible drainage and imbibition processes and to have a capillary pressure curve similar to the reference case of capillary hysteresis (Figures 12a). Figure 12b shows evolutions of the newton iterations per time step for the cases with and without capillary hysteresis. We found that there is almost no difference between the two cases. The total NR (Newton–Raphson) iteration numbers with and without hysteresis are 3646 and 3649, respectively. This implies that nonlinearity induced by the hydrate problem itself prevails over that from capillary hysteresis. Shown in the algorithms, there is no internal iteration during the calculation of capillary hysteresis. Even though we increase the capillary moduli (i.e., α_*p*_ = 2.0 × 10^–8^ Pa^–1^, α_*e*_ = 1.0 × 10^–8^ Pa^–1^, and σ_*Y*_ = 5.0 MPa), there is still little difference from the reference case in computational cost, as shown in Figure 13, where the total NR iterations are 3655. Due to large capillary moduli, from Figure 13c, we find a difference in pressure distribution of the aqueous phase after Step 3, while no difference can be found between the reference case and no capillary hysteresis case because of low capillary moduli (Figure 12c).

### Case 2: Field Application in the UBGH2-6

We apply the implemented code of capillary pressure hysteresis to a gas hydrate deposit in the Ulleung Basin, taking the same geological model as that in Yoon et al.,^18^ restated as follows. Shown in Figure 4, the gas hydrate deposit has an alternating hydrate–mud layer zone of 13 m thickness. A 2D axisymmetric reservoir domain is employed with a vertical well for numerical simulation. The size of the domain is 250 and 220 m in the *x* (radial) and *z* (vertical) directions, respectively. We discretized the domain with 160 × 140 grid blocks, taking the refined grid near the vertical well and the hydrate–mud layer zone. We have no flow boundaries of fluid and heat problems. The initial pressures at the top and bottom are 23.1 and 24.6 MPa, respectively, and the initial temperatures at the top and bottom are 6.37 and 18.63 °C, respectively. They are distributed linearly from top to bottom. The initial porosities of the hydrate and mud layers are 0.67 and 0.45, respectively. The initial hydrate saturation at the hydrate layers is 0.65, while it is zero at the other layers. We have the bulk density of 2650 kg/m^3^ for all layers. Horizontal and vertical permeabilities for the hydrate layer are *k*_*h*_ = 5.0 × 10^–13^ m^2^ and *k*_*v*_ = 1.0 × 10^–13^ m^2^, respectively, while those of the mud layer are *k*_*h*_ = 1.4 × 10^–16^ m^2^ and *k*_*v*_ = 5.5 × 10^–18^ m^2^, respectively. A heat conductivity of 1.45 W/m/°C and a rock specific heat capacity of 1000 J/kg/°C are taken for both the hydrate and mud layers. We take a constant bottom hole pressure for depressurization of gas production. Table 2 shows a production scenario that can induce repeated drainage and imbibition. We used the van Genuchten model of capillary pressure, as used in the previous section.

**Table 2:** Production Scenario for Case 2

steps	time (days)	bottom hole pressure (MPa)
1	0–50	9.0
2	50–51	shut-in

**Figure 14:**
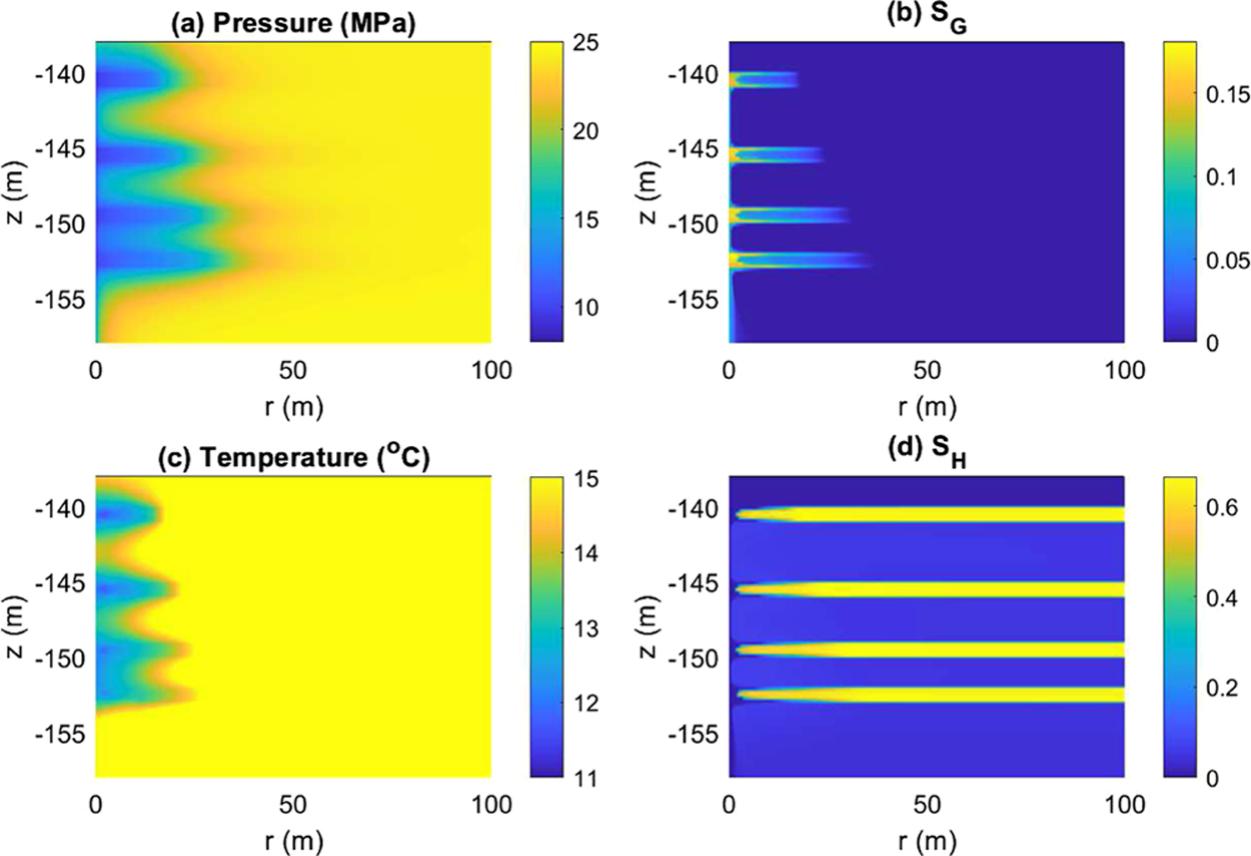
Distribution of pressure, temperature, and saturation after Step 1.

**Figure 15:**
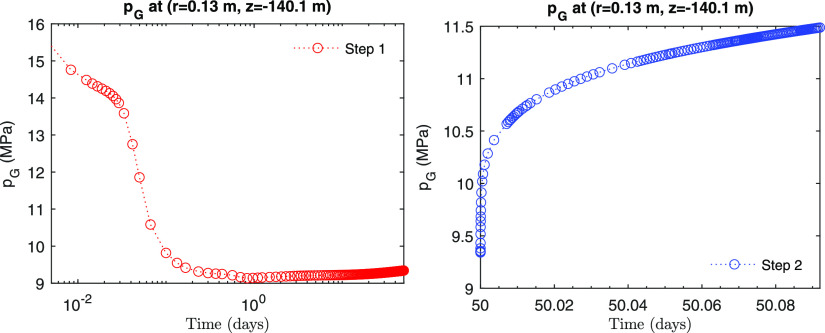
Evolution of pressure at the monitoring point (*r* = 0.13 m, *z* = −140.1 m).

**Figure 16:**
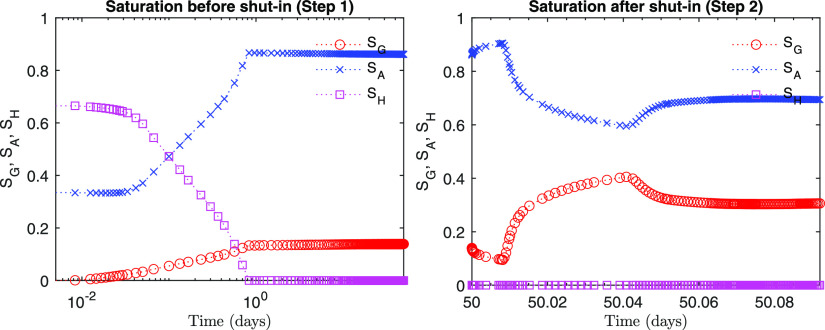
Evolution of saturation at the monitoring point (*r* = 0.13 m, *z* = −140.1 m).

Figure 14 shows distributions of the pressure, temperature, and saturation after Step 1. Dissociation of gas hydrates is induced by depressurization at the well. Gas saturation increases while hydrate saturation decreases overall, and temperature also decreases from the thermodynamic equilibrium condition. After the well is shut in, at Step 2, pressure near the well soars, and saturation changes very rapidly accordingly due to the pressure changes as well as the gravity segregation (Figures 15 and 16). In particular, gas saturation decreases at first during Step 2, while it increases again later. This behavior is an example of the complex physical processes that occur within a very short time scale, induced by well shut-in.

**Figure 17:**
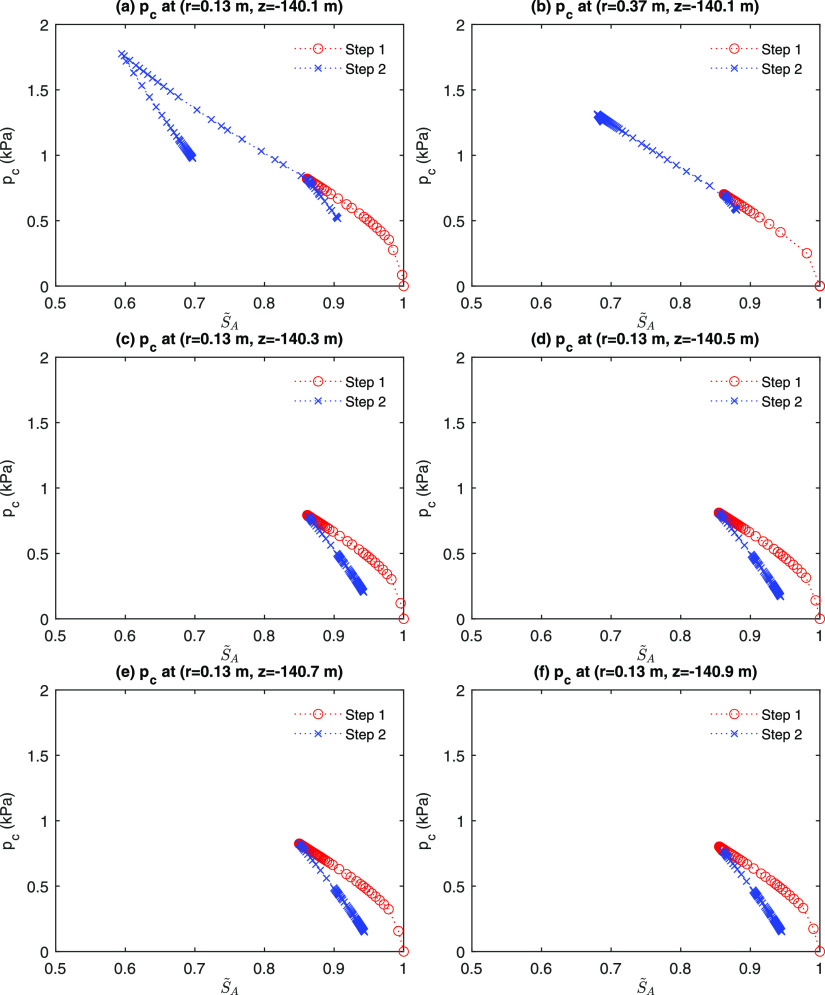
Evolution of capillary pressure at different monitoring points.

Figure 17 shows the evolution of capillary pressure at the different monitoring points near the well. At the monitoring point of (*r* = 0.13 m, *z* = −140.1 m), drainage occurs during depressurization (Step 1), followed by imbibition when the well is shut in in the early time of Step 2, shown in Figure 17a. Note that the first imbibition process does not enter the main imbibition curve. Then, when  increases again, the second drainage process is in the reversible regime, tracing back to the previous imbibition scanning curve. Then, when *p*_*c*_ reaches the main drainage curve, it follows the main drainage curve. Later, as  increases again (the second imbibition), *p*_*c*_ follows the local drainage scanning curve. The monitoring points located in the upper hydrate zone are affected by the gravity segregation, which results in more dynamic changes of  (Figure 17a,b). On the other hand, the areas in the lower hydrate zone experience the first drainage followed by the first imbibition only (Figure 17c–f). We confirm that all of the numerical results are stable.

## Conclusions

We have developed and implemented a code for capillary hysteresis in a gas hydrate simulator. Since the mathematical description of capillary hysteresis in this study is thermodynamically consistent, being well-posed, the corresponding fully implicit numerical algorithm provides numerical stability. Furthermore, the formulation can be applied to several capillary models without restriction. To consider the formation and dissociation of hydrates, gas and aqueous phase saturations are normalized by the total fluid phase saturation. Then, hydrate saturation implicitly affects the evolution of capillary pressure hysteresis as well. The numerical tests support that the developed code for capillary hysteresis yields numerical stability for repeated drainage and imbibition processes and that it can be applied in a field case study such as UBGH2-6 in the Ulleung Basin. To further consider porosity change followed by scaling effects, the code for capillary hysteresis will be extended to coupled flow and geomechanics simulation.
